# High resting energy expenditure, less fat-free mass, and less muscle strength in HIV-infected children: a matched, cross-sectional study

**DOI:** 10.3389/fnut.2023.1220013

**Published:** 2023-09-20

**Authors:** Andrea Franco-Oliva, Beatriz Adriana Pinzón-Navarro, Martha C. Martínez-Soto-Holguín, Ximena León-Lara, Javier Ordoñez-Ortega, Ana Laura Pardo-Gutiérrez, Martha Guevara-Cruz, Azalia Avila-Nava, Alda Daniela García-Guzmán, Laura Guevara-Pedraza, Isabel Medina-Vera

**Affiliations:** ^1^Departamento de Metodología de la Investigación, Instituto Nacional de Pediatría, Ciudad de México, Mexico; ^2^Maestría en Nutrición Clínica, Escuela de Dietética y Nutrición del ISSSTE, Ciudad de México, México; ^3^Servicio de Gastroenterología y Nutrición Pediátrica, Instituto Nacional de Pediatría, Ciudad de México, Mexico; ^4^Tecnologico de Monterrey, Escuela de Medicina y Ciencias de la Salud, Ciudad de México, Mexico; ^5^Institute of Immunology, Hannover Medical School, Hannover, Germany; ^6^Departamento de Infectología Pediátrica, Instituto Nacional de Pediatría, Ciudad de México, Mexico; ^7^Departamento de Fisiología de la Nutrición, Instituto Nacional de Nutrición y Ciencias Médicas Salvador Zubirán, Ciudad de México, Mexico; ^8^Unidad de Investigación, Hospital Regional de Alta Especialidad de la Península de Yucatán, Mérida, Yucatán, Mexico; ^9^Servicio de Oncología Médica, Instituto Nacional de Pediatría, Ciudad de México, Mexico; ^10^Universidad Anahuac, Ciudad de México, Mexico

**Keywords:** HIV-infected, children, adolescents, resting energy expenditure, fat free mass, handgrip strength

## Abstract

**Background and aims:**

Many improvements have been made in the treatment of human immunodeficiency virus (HIV) in pediatric patients; however, challenges remain in terms of achieving normal growth, body composition, and metabolism during treatment, etc. Current nutritional recommendations are based on studies performed in adults, with limited data on the HIV-infected pediatric population. Therefore, this study aimed to compare the resting energy expenditure (REE) of asymptomatic HIV-infected pediatric patients with healthy counterparts and to compare body composition, dietary intake, and physical activity between the two groups.

**Methods:**

This was a cross-sectional study of asymptomatic HIV-infected children who were receiving antiretroviral therapy; the infected group was compared with the uninfected group, matched by age (± 6 months), sex, and body mass index (± 0.5 z-score). Participants were recruited between 2021 and 2022, as outpatients. In both groups, REE was determined by indirect calorimetry and body composition by bioelectrical impedance analysis and hand strength, measured using a hydraulic hand dynamometer.

**Results:**

Seventy-eight participants were enrolled, where *n* = 39 HIV-infected children and *n* = 39 controls, with a mean age of 11.6 ± 3.4 years old. REE was significantly higher in the HIV group (1254.4 ± 334.7 kcal/day vs. 1124.7 ± 321 kcal/day, *p* = 0.013) than in the control group. Fat-free mass (FFM) was lower in the HIV group (28.2 ± 10.5 kg vs. 32 ± 11.2 kg, *p* = 0.001); this trend continued when the index skeletal muscle was evaluated (7.2 ± 1.2 vs. 7.6 ± 1.5, *p* = 0.04). The strength of the dominant hand was also lower in the HIV group (12 (8–18) kg vs. 20 (10.5–26) kg, *p* < 0.0001).

**Conclusions:**

Children with asymptomatic HIV infection have higher REE than their uninfected peers. They also present decreased FFM, skeletal muscle mass index, and muscle strength. These parameters should be considered during nutritional assessment in this population to have a favorable impact on nutritional status and growth.

## 1. Introduction

Resting energy expenditure (REE) refers to the energy expended by an individual in a fasting state, under resting conditions, and in a thermostable environment (1, 2). REE is measured by employing calorimetric methods or evaluated by energy estimating equations. Indirect calorimetry is the most accurate method for measuring REE (3).

Increased REE, protein catabolism, lipodystrophy, and dyslipidemia have been described in human immunodeficiency virus (HIV)-infected adults (4). A meta-analysis that included studies in the adult population living with HIV reported that the mean of REE per fat-free mass (FFM) was significantly higher in 732 HIV-positive subjects than in 340 control subjects, with a mean of 11.93 kJ/kg (95% CI: 8.44,15.43 kJ/kg) (5). In addition, the energy requirements of asymptomatic untreated HIV-infected adults are 7–15% higher than those of uninfected controls such that maintaining body weight in this population requires increasing energy intake by ~10% (6). In HIV-infected children, the energy balance and REE determination are crucial, as there is a high prevalence of failure to thrive in this population (7). A mean REE of 57 ± 7 kcal/day/kg has been reported in HIV-infected children aged 2–11 years (8). Nevertheless, there is currently limited evidence on the energy requirements of HIV-infected pediatric patients, and current nutritional recommendations are based on studies performed in adults (9). In pediatrics, it is particularly important to cover energy needs to continue with growth (6).

Therefore, this study aimed to compare the REE of asymptomatic HIV-infected pediatric patients with that of healthy controls. We hypothesized that the group with HIV has 10% more REE than the control group. Our secondary objective was to compare the body composition, handgrip strength, dietary intake, and physical activity of HIV-infected pediatric patients with those of the control group.

## 2. Methods

### 2.1. Study design

We performed a cross-sectional study of HIV-infected children and uninfected children as a control group matched by gender, age (± 6 months), and body mass index for age (BMI-for-age) (± 0.5 z-score). Participants were recruited between 2021 and 2022 as outpatients in the Infectious Disease Department of the Instituto Nacional de Pediatría, a third-level pediatric hospital in Mexico City, México. The control group was recruited from schools nearby that were healthy.

Patients aged 6–18 years were included; in the HIV-infected group, only patients who were receiving highly active antiretroviral therapy were included. Participants in both groups were excluded if they were taking anabolic agents, stimulants, or any drugs known to alter metabolism. They were also excluded if they had diabetes; an active opportunistic infection; malignancy; or hepatic disease, including chronic hepatitis, pulmonary disease, and severe immune compromise defined by CD4+ count <200 cells/mm^3^. This was an exclusion criterion because severe immune compromise is generally accompanied by infectious processes that increase REE (10).

### 2.2. Clinical evaluations and anthropometric parameters

Parents or primary caregivers were asked about their personal and family history, as well as the presence of symptoms at the time of evaluation. The date of diagnosis was obtained from patients' medical records. Participants were weighed on a calibrated digital scale (SECA 813; Seca GmbH&Co., Hamburg, Germany), and height was measured with an ultrasonic stadiometer (InLab S50; InBody Co., Seoul, Korea). Waist, hip, thigh, calf circumferences, and mid-upper arm circumference (MUAC) were measured with a tape measure (SECA 201; Seca GmbH&Co., Hamburg, Germany). All measurements were taken with the patients standing up. Waist circumference was measured with the arms crossed in front of the chest; the measurement was taken between the lower edge of the 10th rib and the iliac crest. Hip circumference was measured by placing a tape measure at the biggest protuberance of the buttock; thigh circumference was measured with the legs separated, and the tape was wrapped around the mid-point between the hip bone and the knee bone. Calf circumference was measured with the arms by the side of the body; the measurement was taken at the biggest protuberance of the calf; Finally, MUAC was measured with the arms by the side of the body; the measurement tape was positioned halfway between the acromion and the radius measurements (11, 12).

### 2.3. Resting energy expenditure

REE was measured by using the ReeVue^TM^ device, (indirect calorimeter, Korr Technologies Inc, Salt Lake City, Utah, USA). Measurements were performed in a room with a stable room temperature (21°C) and a quiet environment. O_2_ flows were measured directly with a mask and an 18 mm diameter flowmeter. Flowmeter calibration was performed before each test. All measurements were performed in the morning (between 08:00 and 09:30), with 8 to 12 h of fasting. Participants were instructed to refrain from exercise for at least 12 h (vigorous and resistance exercise for 24 h prior to testing). Upon arrival at the clinic, the participants sat for ~20–30 min while the study was explained to them; they signed consent and assent letters, and their medical history was taken. Participants were tested in the supine position for a period of at least 20 min with minimal movement, ensuring that each individual was physically comfortable and in the proper position for measurements.

### 2.4. Nutritional status and body composition assessment

Nutritional status was assessed by the z-score of BMI-for-age and height-for-age, according to the classification and values established by the CDC and WHO. To obtain the z-score of BMI-for-age, the PediTools program was used (13), in which data such as gender, date of birth, date of evaluation, weight, and height were entered. The reference points were classified according to the WHO, where < −3 SD = severe malnutrition, −3 to −2 SD = moderate malnutrition, ≥ −2 to 1 SD = standard, >1 to <2 SD = overweight, and >2 SD = obesity (14). The height-for-age indicator was evaluated using the AnthroPlus software (15), in which the height/age was obtained using the same data. The cut-off points were classified according to the WHO: 1.99 to −1.99 SD standard height, <-2 SD short height, and >2 SD tall height (14). Body composition was assessed by using a multifrequency bioimpedance device, employing bioelectrical impedance analysis (BIA) (InBody S10^®^, InBody Co., Ltd., Seoul, Korea) with the standard technique; BIA's internal equation was used. Measurements were performed with the patient in a supine position, with the arms separated from the trunk by ~30 degrees and the legs separated by ~45 degrees; there was no contact with the metal frame of the bed, and the room temperature was ambient. The patients had to lie in position for 5 min and were not allowed to eat or make any major physical effort in the preceding 8 h; they were also not allowed to drink in the preceding 3 h. Body weight and height were entered into the device. The area where the electrodes were to be placed was cleaned first with alcohol and then with electroconductive wet wipes for the use of impedance equipment; the electrodes were placed on both the hands and the feet, according to the manufacturer's instructions (InBody Co). The electrodes were kept in a sealed bag to protect against heat; the machine was calibrated before use with a circuit of known impedance, as per the manufacturer's guidelines. A standardized healthcare professional performed the measurements using the same device to avoid interobserver and inter-device variability. Phase angle at 50 kHz was reported, and the following formula was used to determine it [Arc tangent (Xc/R)] × (180/Π). The skeletal muscle mass index (SMI) was calculated by dividing skeletal muscle mass (kg) by the square of the height (m^2^), and the impedance ratio was calculated as the quotient of Z at 250 kHz between Z at 5 kHz. Additionally, the total body water (TBW) = (height^2^/ impedance (R), skeletal muscle mass (SMM) = FFM (right arm + left arm + right leg + left leg)/0.75, and body cell mass (BCM) = intracellular water (ICW) + proteins were estimated by internal equations of BIA.

### 2.5. Handgrip strength evaluation

Handgrip strength, an indirect indicator of muscle function, was measured with a Lafayette hydraulic hand dynamometer (Jamar model J00105 Lafayette Instrument Company, USA 90k g capacity and 727 g weight). The measurement of grip strength was performed on the dominant hand in triplicate, and the best measurement was recorded. The posture for measuring the grip strength involved standing with legs straight and weight bearing balanced on both feet; the feet were positioned shoulder-width apart, shoulders were adducted and neutrally rotated, elbows were flexed to 90°, forearms were in a neutral position, and the wrists were between 0° and 30° of dorsiflexion and between 0° and 15° of ulnar deviation (16).

### 2.6. Routinary serum biochemical parameters

The most recent laboratory results of the following biochemical parameters were obtained from the medical record: viral load, CD4+ cell count, CD8+ cell count, lipid profile, hemoglobin, transaminases, albumin, glucose, creatinine, and glomerular filtration rate. These labs were routinely requested by the treating service as part of the follow-up consultation (only for the HIV-infected group).

### 2.7. Physical activity

Body movement produced by skeletal muscles was estimated using the Physical Activity Questionnaire for Children (PAQ-C) for children aged 8–14 years and the Physical Activity Questionnaire for Adolescents (PAQ-A) for children > 14 years. The questionnaires consist of questions structured to discern low (score 1) to high (score 5) physical activity during the last 7 days (17). All score items were then summed and divided by the number of questions to yield the final activity summary score, which was finally classified as follows: > 2.151 was classified as active, meeting 60 min of moderate-to vigorous-intensity physical activity (MVPA), and < 2.151 was classified as not active (18).

### 2.8. Dietary evaluation

To assess dietary intake, 24-h multi-step recall was performed with FAO methodology (19). The information was analyzed using the NIH software, ASA24 (20). In addition to counting calories, macronutrients, and micronutrients, recommendations for target amounts of food groups, calories, macronutrients, and micronutrients by gender and age were obtained from each participant using the following sources: The 2015–2020 Dietary Guidelines for Americans https://www.dietaryguidelines.gov and https://www.dietaryguidelines.gov and the Dietary Reference Intakes https://www.nal.usda.gov/fnic/macronutrients and https://www.nal.usda.gov/fnic/macronutrients. Therefore, the diet analysis was not only compared between the groups but also with the recommended intake by gender and age. The questionary “Index of global food quality” (21) was also evaluated to assess the overall diet quality. This index is a food frequency survey with 12 variables: 5 healthy foods, 4 unhealthy foods, and 3 main meals. Each variable was scored from 1 (less healthy) to 10 (recommended by the Ministry of Health). The total scores were used to classify diets as healthy (90–120), in need of change (60–89), and unhealthy (<60).

### 2.9. Statistical analysis

Sample size calculation was carried out, where it was considered that the REE of pediatric patients with HIV infection is >10% (6) when compared with that of uninfected children. Furthermore, a type 1 error (α) of 0.05 and a type II error (β) of 0.2 were considered. A sample of 40 per group was obtained. 3 To compare quantitative variables between the HIV-infected group and the uninfected group, including REE and body composition parameters (lean mass, fat mass, and phase angle), as well as dietary assessment variables (kilocalories, grams of protein, carbohydrate, fat, and micronutrients), Student's *t*-test for paired samples or the Wilcoxon test was performed, depending on the distribution of the variable. To compare the categorical variables, an Xi^2^ analysis was performed. An analysis of covariance (ANCOVA) was also performed to examine the differences in REE by groups (HIV-infected vs. control group). This analysis accounted for FFM, FM, height, body weight, and physical activity, each separately as a covariate but also as a set of variables: height, FFM, and FM for model A and FFM, FM, height, and physical activity for model B. FFM and FM were included in the models to analyze the weight of the body composition, height was included because it differed between groups, and physical activity was included because it is an important variable that can influence body composition. A significant *p-*value was established as ≤ 0.05. The data were analyzed using SPSS (25 version, SPSS Inc, Chicago, IL) and GraphPad Prism (version 9.0, GraphPad Software, La Jolla California, USA).

### 2.10. Ethical statement

The protocol was approved by Instituto Nacional de Pediatría Research and Ethics Committees with number 2020/026, officially registered at the Office for Human Research Protections of the NIH (http://ohrp.cit.nih.gov/search/search.aspx), with numbers IRB00013674 and IRB00013675. All the subjects' information was handled confidentially. Each participant and parents or primary caregivers signed a written informed assent and consent form, respectively, before enrollment.

## 3. Results

### 3.1. Demographics

Forty pediatric patients with HIV were evaluated. As the patients in the HIV-infected group entered the study, a paired search for uninfected children was performed. One patient was removed from the HIV-infected group because he presented a z-score for BMI-for-age of – 4 SD; it was not possible to find a participant with this characteristic, without pathology. Therefore, the results of 39 patients in the HIV-infected group paired with 39 uninfected patients are reported. All HIV-infected children included in the analysis were asymptomatic at the time of evaluation and were receiving highly active antiretroviral therapy; 49% of HIV-infected children were on abacavir, lamivudine, lopinavir, and ritonavir, and 32% were on bictegravir, emcitarabine, tenofovir, and alafenamide. The remaining patients were receiving antiretroviral therapy, which was guided by resistant patterns, where highly active antiretrovirals such as dolutegavi, raltegravir, and efavirenz were included in follow-up as outpatients by the pediatric infectious disease department. All HIV-infected patients had vertically transmitted infection and CD4+ levels above 600 cells/mm^3^, and 82% (*n* = 32) of the patients had undetectable viral load (Supplementary Table 1). When compared between groups, a high prevalence was observed in boys (64%) in each group, and there was no significant difference in age and diagnosis of nutritional status evaluated by the BMI-for-age between groups. However, there were differences in almost all anthropometric variables, except for waist circumference. Additionally, it was observed that 39.5% (*n* = 15) of the children in the HIV-infected group showed stunting, while only 5.1% (*n* = 2) in the control group showed stunting (Table 1).

**Table 1:** Baseline characteristics of the participants.

**Variables**	**HIV-infected group *n =* 39**	**Control group *n =* 39**	***p***
Age, years	11.6 ± 3.5	11.6 ± 3.4	0.934
Gender: Girls/Boys, *n* (%)	14 (36)/25 (64)	14 (36)/25 (64)	1
Body weight, kg	36.4 ± 14.4	40.3 ± 14.9	0.001
Height, cm	138.3 ± 18.2	146.1 ± 17.6	< 0.0001
Nutritional status^*^, ***n*** **(%)**			
Undernourishment	2 (5.2)	2 (5.2)	1
Normal	32 (82.1)	32 (82.1)	
Overweight	5 (12.9)	5 (12.9)	
**Height-for-age**, ***n*** **(%)**			0.001
Normal height	24 (60.5)	36 (92.3)	
Short height	15 (39.5)	2 (5.1)	
Tall height	0 (0)	1(2.6)	
**Anthropometric assessment**			
BMI-for-age	−0.5 ± 1.17	−0.009 ± 1.13	0.490
Height-for-age, z-score	−1.30 ± 0.9	−0.10 ± 1.2	< 0.0001
Waist circumference, cm	65.2 ± 10.7	66 ± 9.8	0.424
Waist/height index	0.47 ± 0.05	0.45 ± 0.05	0.008
Hip circumference, cm	72.7 ± 12	76.9 ± 13.4	< 0.0001
Thigh circumference, cm	38.2 ± 6.6	40.3 ± 8.1	0.014
Leg circumference, cm	27.1 ± 5.3	29.6 ± 5.1	0.002
MUAC, cm	21.4 ± 4	22 ± 4.2	0.035

Data are shown as mean ± standard deviation and as frequency and percentage. Student's t-statistical analysis for paired samples and Xi^2^ of trend. p < 0.05. kg, kilograms; cm, centimeters; m, meter; and MUAC, mid-upper arm circumference. ^*^Evaluated by the BMI-for-age indicator.

Furthermore, we observed a strong correlation between anthropometric indicators and BIA indicators, as well as a high positive correlation between body weight and FFM, SMM, BCM, and TBW (Supplementary Figure 1).

### 3.2. Comparison of resting energy expenditure

**Figure 1:**
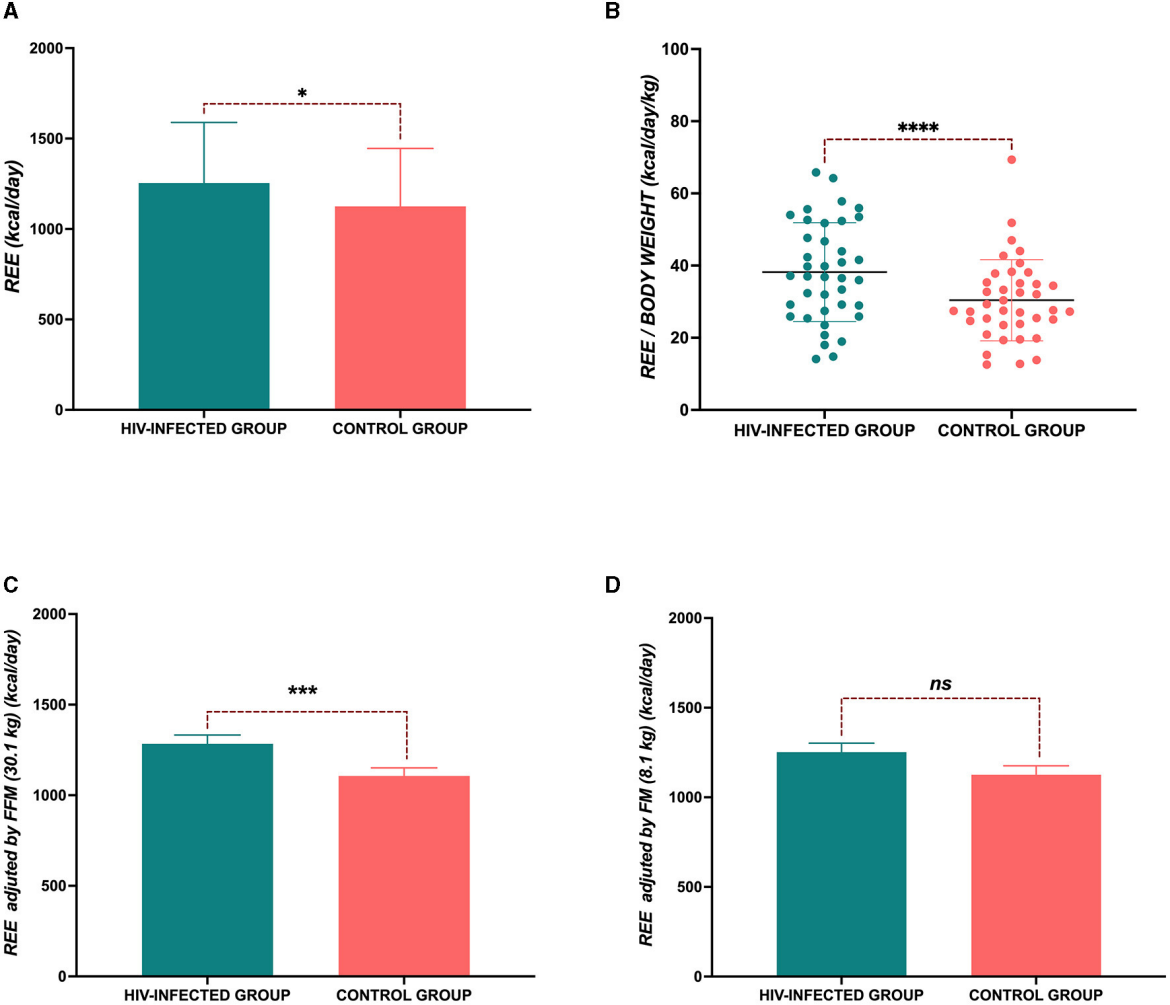
Resting energy expenditure (REE). **(A)** Total REE; **(B)** REE per kg of body weight; **(C)** REE adjusted by fat-free mass (FFM); and **(D)** REE adjusted by fat mass (FM). *P-*value: ^*^p < 0.05; ^****^p < 0.0001.

In the HIV-infected group, there was an increase in REE compared with the control group (1254.4 ± 334.7 kcal/day vs. 1124.7 ± 321 kcal/day, *p* = 0.013) (Figure 1A). Additionally, we evaluated the REE by body weight, and we also observed an increase in REE by kg (38.1 ± 13.6 kcal/day/kg vs. 30.4 ± 11.2 kcal/day/kg, *p* < 0.0001) (Figure 1B). When adjusted for FMM, this upward trend continued (Figure 1C). We also adjusted for FM, but no differences were observed (*p* = 0.083) (Figure 1D). Furthermore, we calculated the percentage of increase in REE in the HIV-infected group compared with the control group and observed an increase of 8.2% (CI 95% 3.1–20.9%) in the total REE, 22.5% (CI 95% 11.6–35.2%) more per kilogram of body weight.

In the unadjusted analyses, we observed differences in REE between groups. However, after accounting for relevant covariates in the analysis, including body weight, REE was higher in the HIV-infected group 1269.7± 47.7 [SE] kcal/day vs. 1117.3 ± 45.4 [SE] kcal/day, *p* = 0.020. The same high REE trend was observed in the HIV-infected group when covariates such as height and FFM were analyzed, but when we analyzed FM as a covariate, we did not observe any statistical differences. Furthermore, we analyzed a model where FFM, FM, and height were included (Table 2). Sensitivity analysis was also performed in the HIV-infected group to compare REE between patients who had an undetectable viral load (<40 copies/ml of blood) (*n* = 32) and those who had >40 copies/ml of blood (*n* = 7); no significant difference was observed [1217 (968–1570) kcal/day vs. 1238 (1109–1498) kcal/day, *p* = 0.781]. Similarly, another analysis was performed, eliminating those with >40 copies/ml of blood, and only 32 patients in the HIV-infected group were compared with patients in the control group (*n* = 32). The HIV-infected group had higher REE (1245 ± 355 kcal/day vs. 1122 ± 331 kcal/day, *p* = 0.02).

**Table 2:** ANCOVA analysis comparing REE between groups.

**ANCOVA adjusted by**	**Adjusted REE (kcal/day) Mean** ±**SE**		**Difference in adjusted mean change (HIV-infected group–Uninfected group)**		
	**HIV-infected group, (*****n** =* **39)**	**Uninfected group, (*****n** =* **39)**	**Mean** ±**SE**	**95% CI**	***P*** **-value**
Body weight (38.4 kg)	1269.7 ± 47.7	1117.3 ± 45.4	152.4 ± 64	24.8, 279.9	0.020
Height (142.2 cm)	1286.1 ± 49.5	1098 ± 48.2	188 ± 69.6	49.3, 326.8	0.009
Fat-free mass (30.1 kg)	1284.5 ± 48.3	1106 ± 45.4	178.5 ± 64.8	49.3, 307.7	0.007
Fat mass (8.1 kg)	1252.5 ± 50	1126 ± 50	125.9 ± 71	−17, 268.8	0.083
Physical activity (PAQ=2.3)	1252.5 ± 52	1126.5 ± 51	126 ± 75	−23.7, 275	0.098
Model A	1276.5 ± 49.4	1102 ± 54.1	173.9 ± 71	32, 315.8	0.017
Model B	1280.1 ± 50	1099 ± 49	181.1 ± 72.4	36.7, 325.5	0.015

A: Model adjusted by FFM = 30.1 kg, FM = 8.1 kg, and height = 142.2 cm. B: Model adjusted by FFM = 30.1 kg, FM = 8.1 kg, height = 142.2 cm, and PAQ = 2.3.

### 3.3. Body composition, phase angle, and handgrip strength

**Figure 2:**
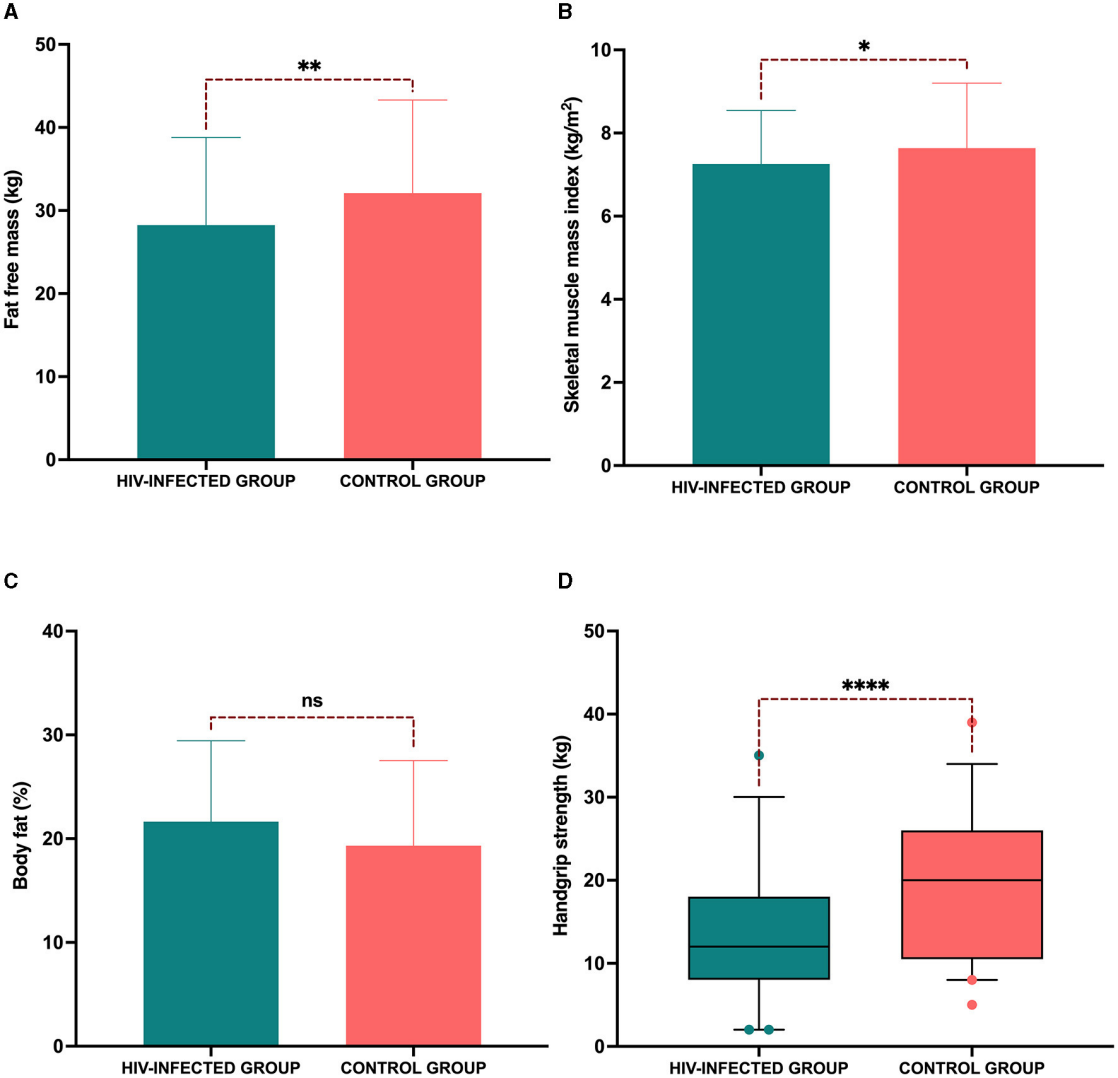
Reduced fat-free mass and muscle strength in the HIV-infected group. **(A)** Fat-free mass; **(B)** skeletal muscle mass index; **(C)** percentage of body fat; and **(D)** handgrip strength in the dominant hand. *P*-value: *p < 0.05; **p < 0.001, ****p < 0.0001.

When we analyzed the body composition between groups, significant differences were observed in TBW, FFM, and BCM. However, no significant differences were observed in or in the area of visceral fat and total phase angle. Interestingly, the phase angle of the lower extremities was higher in the control group (Table 3). Regarding FFM, less mass was observed in the HIV-infected group (28.2 ± 10.5 vs. 32 ± 11.2, *p* = 0.001). This trend continued when index skeletal muscle was evaluated (7.2 ± 1.2 kg/m^2^ vs. 7.6 ± 1.5 kg/m^2^, *p* = 0.04). When the strength of the dominant hand was compared (12 [8–18] vs. 20 [10.5–26] kg, *p* < 0.0001), it was lower in the HIV-infected group. However, the percentage of body fat mass (21.6 ± 7.7 vs. 19.3 ± 8.2 %, *p* = 0.131) did not present any differences between groups (Figure 2).

**Table 3:** Body composition measured by bioelectrical impedance of groups.

**Variables**	**HIV-infected group *n =* 39**	**Control group *n =* 39**	***p***
Intracellular water, L	12.8 ± 4.8	14.4 ± 5.1	0.003
Extracellular water, L	7.8 ± 2.8	8.7 ± 2.9	0.004
Total body water, L	20.7 ± 7.7	23.2 ± 8	0.003
Proteins, kg	5.5 ± 2.1	6.2 ± 2.2	0.003
Minerals, kg	1.9 ± 0.7	2.3 ± 0.8	< 0.0001
Soft lean mass, kg	26.6 ± 9.9	30 ± 10.4	0.002
Skeletal muscle mass, kg	14.7 ± 6.3	16.9 ± 6.8	0.003
Body cell mass, kg	18.3 ± 7	20.8 ± 7.5	0.002
Visceral fat area, cm^2^	33.3 ± 24.6	30.8 ± 20.1	0.443
Total phase angle, °	5.2 ± 0.7	5.4 ± 0.8	0.170
Right arm phase angle, °	4.9 ± 0.5	4.7 ± 0.6	0.171
Left arm phase angle, °	4.9 ± 0.6	4.5 ± 0.6	0.006
Trunk phase angle, °	6 ± 1.0	5.9 ± 1.0	0.315
Right leg phase angle, °	5.7 ± 1.1	6.3 ± 1.0	0.004
Left leg phase angle, °	5.5 ± 0.9	6 ± 1.0	0.001
Impedance ratio 250/50 kHz, °	0.89 ± 0.01	0.89 ± 0.00	0.132

Data are shown as mean ± standard deviation. Student's t-statistical analysis for paired samples. P < 0.05. L: liters; kg: kilograms; %: percentage; and cm^2^: square centimeters.

### 3.4. Diet analysis and diet quality

**Figure 3:**
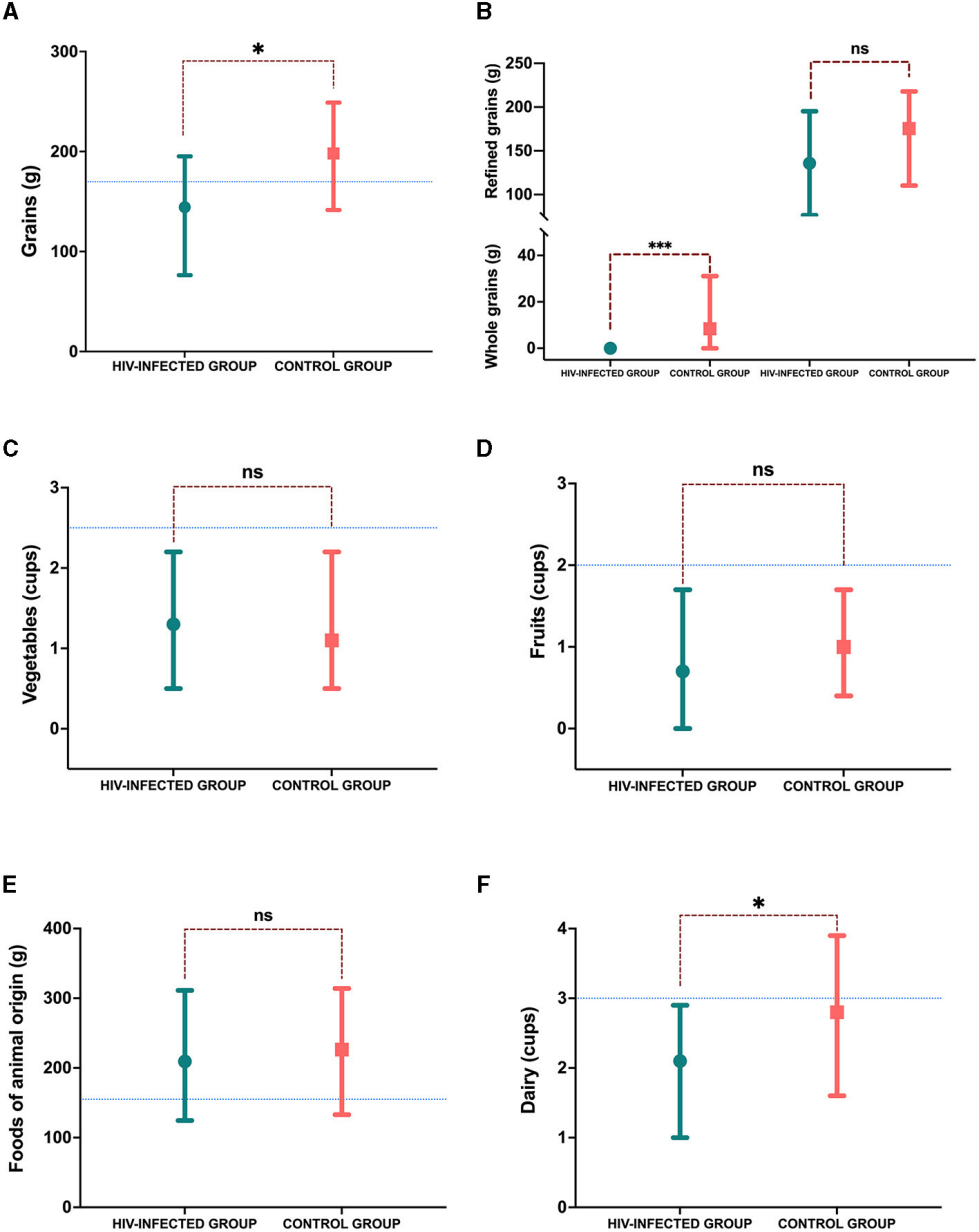
Consumption of food groups. **(A)** Grains consumption in grams; **(B)** grain consumption divided by whole and refined in grams; **(C)** vegetable consumption in cups; **(D)** fruit consumption in cups; **(E)** foods of animal origin in grams; and **(F)** dairy consumption in cups. The blue line marks the median recommendation for sex and age; *p*-value: *p < 0.05.

In the analysis of the participants' diet, the HIV-infected group showed a significant reduction in energy consumption compared to the control group (1660 ± 557 kcal vs. 2092 ± 591 kcal, *p* = 0.001), with energy being lower in the HIV-infected group. However, in the analysis of energy consumption per kg of body weight, we did not observe any statistically significant differences, although the trend showed lower calorie consumption in the HIV-infected group (48.2 ± 21 kcal/kg vs. 56.9 ± 18.6 kcal/kg, *p* = 0.06). Regarding the percentage of macronutrients referring to the caloric value total, no significant differences were observed in the consumption of carbohydrates (46.4 ± 8.3% vs. 45.1 ± 8.7%, *p* = 0.495), proteins (21.2 ± 6 % vs. 20.8 ± 5.7%, *p* = 0.773), or fats (32.2 ± 7 % vs. 34.6 ± 6.3 5, *p* = 0.152) between the HIV-infected group and the control group. However, in the food consumption analysis, we observed that the HIV-infected group showed lower grain consumption (144.3 [76.4–195.2] g vs. 198.1 [141.5–249] g, *p* = 0.029). As for the recommendation considering age and gender (169.8 [169.8–198.1] g of grains) (Figure 3A), the median of the control group was above the recommendation, but that of the HIV-infected was below the recommendation. When analyzed by the type of grain consumed, the consumption of whole grains was null in the HIV-infected group (0 [ 0–0] g vs. 8.4 [0–31.1] g, *p* = 0.003), and in the consumption of refined grains was higher (135.8 [76.4–195.2] g vs. 175.4 [110.3–217.9] g, *p* = 0.106) (Figure 3B). Vegetable consumption did not differ between groups (1.3 [0.5–2.2] cups vs. 1.1 [0.5–2.2] cups, *p* = 0.992), and the consumption of neither group reached the recommendation (2.5 [2.5–3] cups) (Figure 3C). Fruit consumption did not differ between groups (0.7 [0–1.7] cups vs. 1 (0.4–1.7] cups, *p* = 0.472); the consumption of both groups was below the median recommendation (2 [1.5–2] cups) (Figure 3D). The consumption of food of animal origin was above the median of recommendation for both groups (155.6 [141.5–169.8] g), but there was no difference in the consumption between groups (209.4 [124.5–311.3] g vs. 226.4 [133–314.1] g, *p* = 0.839) (Figure 3E). Finally, dairy consumption was higher in the control group (2.1 [1–2.9] cups vs. 2.8 [1.6–3.9] cups, *p* = 0.015) than in the HIV-infected group; the median of recommendation for dairy was 3 [3–3] cups (Figure 3F).

**Figure 4:**
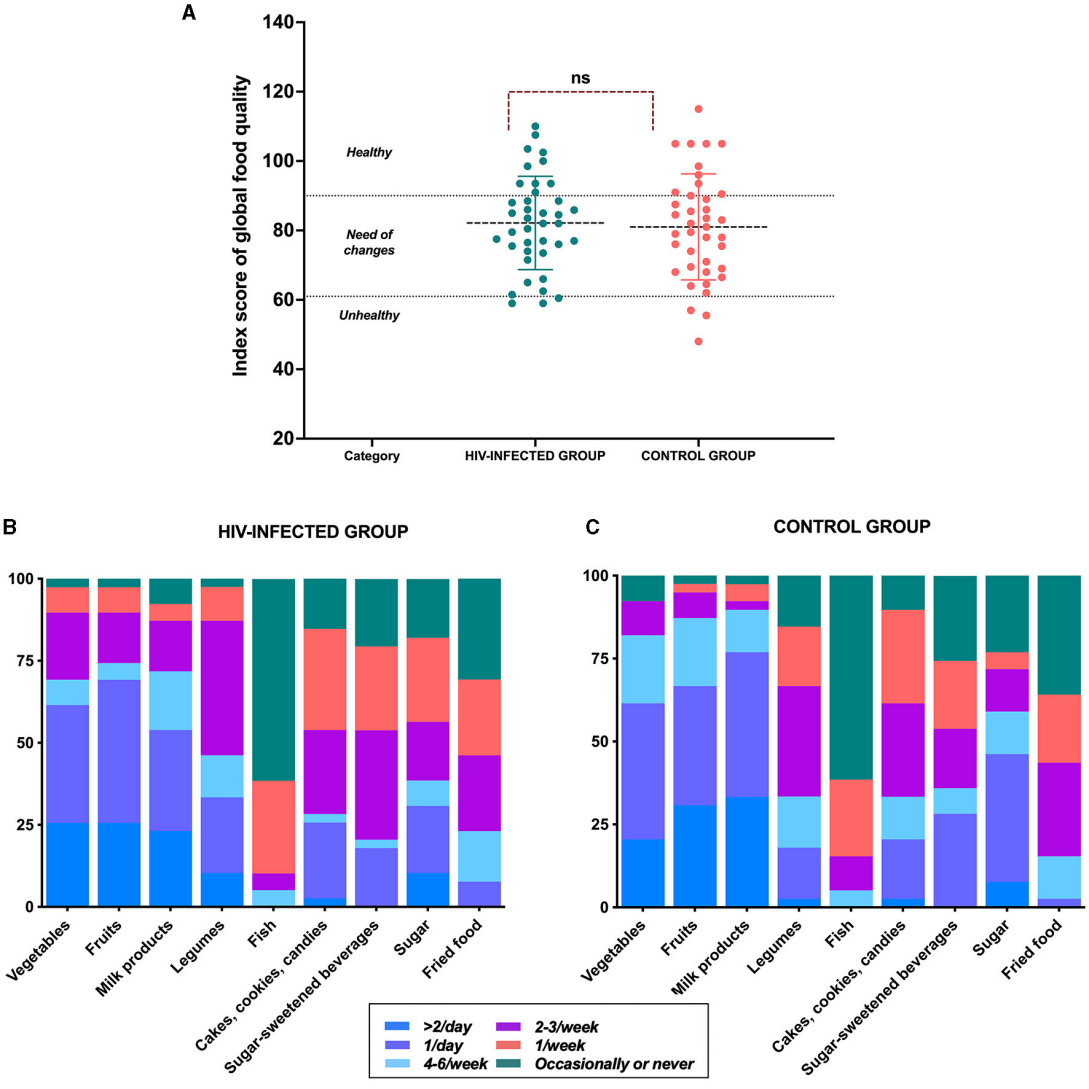
Index score of global food quality. **(A)** Index score of global food quality comparison; **(B)** frequency of consumption of food groups in the HIV-infected group, and **(C)** control group.

As for micronutrient consumption, calcium consumption was lower in the HIV-infected group (887.5 ± 454 mg vs. 1214.2 ± 484.8 mg, *p* = 0.004); the recommendation for calcium was 1300 [1300–1300] mg. Vitamin D consumption was lower in both groups, based on the recommendation (600 600–600] IU), and it did not differ between groups (296.6 ± 244.2 IU vs. 296.2 ± 152 IU, *p* = 0.994). Fiber consumption was also lower in both groups, based on the recommendation (31 [26–31] g), and the consumption did not differ between groups (12 [9–18] g vs. 16 [13–22] g, *p* = 0.079). However, sodium consumption was higher in the control group (2548 [1928–3363] mg vs. 3238 [2384–4076] mg, *p* = 0.014), and the consumption of both groups was above the recommendation (1800 [1800–2300] mg). When the diet quality index was analyzed, no significant difference was observed in the average score between both groups in terms of the consumption of other micronutrients such as vitamins. (82.1 ± 13.4 vs. 81 ± 15.2, *p* = 0.725). Using the stratification of the index, the percentage of participants in the “healthy” stratum was 25.6% in the HIV-infected group and 28.2% in the control group. In the “need for changes” stratum, it was 66.7 and 64.7% in the HIV-infected and control groups, respectively. Finally, the “unhealthy” stratum was 7.7% in both groups (Figure 4A). The frequency of consumption of foods from the index was highly similar between groups. This highlighted that in both groups, the consumption of fish was higher, with the frequency of “occasionally or never” being 61.5% in both groups. It stood out for the consumption of legumes, as 23.1% of the participants in the HIV-infected group ate legumes once a day, whereas in the control group, the frequency was 15.4% (Figures 4B, C).

### 3.5. Physical activity

According to the physical activity questionnaires, there were no differences in the scoring (2.2 ± 0.9 vs. 2.4 ± 0.6, *p* = 0.226) between groups. When the strata analysis was performed, 30.8% of children in the control group were not active (<60 min of MVPA level), and 51.3% of children in the HIV-infected group were not active (*p* = 0.066) (Supplementary Figure 2).

## 4. Discussion

Our results showed that REE was significantly higher among children diagnosed with HIV infection than children in the control group, matched by BMI, gender, and age. To our knowledge, this is the first study to compare REE paired with these variables in the pediatric population. Although the WHO recommends a 10% increase in REE in HIV-infected pediatric patients (22), the available studies referenced are from the adult population (10, 23–26). However, Henderson et al. reported no significant differences between 29 pediatric patients with HIV (2–11 years) and 9 uninfected pediatric patients (57 ± 7 kcal/kg/day vs. 50 ± 13 kcal/kg/day) (8), although the sample size of the control group was a limitation in their study.

Similar findings have been reported in the adult population. Grinspoon et al. (27) evaluated 33 HIV-infected premenopausal women and 26 healthy premenopausal women who were part of the control group; the women were matched for body weight. The researchers concluded that REE was higher in the HIV-infected women than in the control women (1624 ± 329 vs. 1437 ±145 kcal/d), *p* = 0.0096); they also concluded that FFM was the main determinant of REE in the HIV-infected women. Supporting these findings, Lane et al. (28) also evaluated REE in asymptomatic and newly diagnosed HIV-infected women (35 ± 7 years), who were compared with a control group matched by age, BMI, and FFM. They observed that REE was higher in the HIV+ group when the REE adjusted for differences in body composition (31.4 ± 1.75 vs. 29.9 ± 2.5 kcal/kg FM and FFM, *p* = 0.04) in the early stages of the disease. Likewise, in a comparative study carried out by Hommes et al. (23), it was observed that asymptomatic HIV-infected patients had 8% (*p* < 0.05) higher REE rates than healthy control subjects; suggesting that HIV infection affects host metabolism in the early asymptomatic stage.

The proposed mechanisms for elevated REE in people with HIV may be related to viral load, CD4+ cell count, the use of antiretroviral drugs, body composition, hormones, and proinflammatory cytokines (4). An increase in REE can lead to wasting syndrome in patients, so comprehensive evaluation of pediatric patients with HIV is extremely important, ideally in situations where REE measurement by indirect calorimetry is accessible. Otherwise, it must be taken into account that the use of REE prediction equations could present an estimation bias because there are no predictive equations for pediatric patients with HIV, and the existing equations are based on a healthy population (29). Furthermore, a great clinical variety exists among subjects, and the lack of inclusion of muscle mass as a variable in the equations must also be taken into account. Thus, future research could take into account the design of an REE prediction equation in a pediatric population with HIV to minimize prediction bias.

Interestingly, we found that children with HIV had lower FFM than the control group. Several studies have been conducted on body composition in HIV-infected patients using multifrequency BIA; this technique is non-invasive, safe, and portable and measures body compartments such as FFM, FM, and TBW (intracellular and extracellular water). These studies have reported a reduction in body weight, total body potassium, subcutaneous FM, and SMM, as well as a disproportionate reduction in BCM relative to weight reduction in HIV-infected patients; a reduction in BCM has also been reported to occur in the early stages of the disease (30).

These alterations in body composition can be explained by the presence of proinflammatory cytokines, particularly in high concentrations of TNF-α, which is involved in the degradation of protein because it can inhibit the secretion of the hormone testosterone. Testosterone regulates muscle mass, affects the sensitivity of tissues to hormonal signaling, and directly stimulates the degradation of proteins and FFM (31, 32). Likewise, it has been documented that people infected with HIV experience changes at multiple levels of the hypothalamic-pituitary-adrenal axis, which can cause elevated cortisol levels (33). This alteration, being a potent catabolic pathway, could also increase protein degradation rates and slow down protein synthesis rates, especially in muscle mass; this is why it is implicated in muscle wasting associated with HIV infection (34).

These alterations might affect muscle characteristics and functions such as strength. In this sense, our results showed that in the HIV-infected group, there was a significant decrease of −11% (95% CI: −18, −3%) in handgrip strength, measured by dynamometry; this could be explained by muscle wasting, which reduces muscle mass and strength and increases fatigue (35). In fact, it has been reported that handgrip strength has a greater influence on muscle function than muscle mass (36). In addition, we found a strong positive association between FFM and handgrip strength in the HIV-infected group (*r* = 0.918, *p* = 0.0001); a similar trend was observed in the control group but to a lesser magnitude (*r* = 0.846, *p* = 0.0001). However, other studies have not reported any differences in muscle strength between children who acquired HIV perinatally and children who were HIV-uninfected (37).

Another important finding was in the diet analysis, where we observed that calorie intake was significantly lower in the HIV-infected group than in the control group, even with a higher REE, which is a source of alert. In addition to this, we observed that the quality of the diet in both groups is incorrect for their age and gender. It is important to emphasize that a higher REE and lower FMM in patients with HIV are impressive because they can trigger a negative balance of energy. If patients do not consume adequate energy in their diet according to their demands, they could develop macronutrient and micronutrient deficiencies, and they could also accelerate immunodeficiency development and the appearance of opportunistic infections, thereby increasing the risk of mortality (2). In addition, in pediatric patients, high REE can cause inadequate growth and development (6), so it is essential to determine the adequate nutritional needs of these patients. Furthermore, energy imbalance is more serious in children than in adults because a high proportion of energy is required for growth in healthy children and for catch-up growth in children recovering from an opportunistic infection, which can consequently change their REE. Although factors that contribute to the total daily energy expenditure include physical activity, growth, and diet-induced thermogenesis, these are not accounted for in the REE measurement, and the variation in the results of energy expenditure measurements is likely due to differences in dietary intake, nutritional status, physical activity, severity of illness, and opportunistic infection (38).

Although the phase angle does not contribute to changes in REE, having higher REE and not being able to meet caloric requirements may be associated with a lower phase angle, reflected in reduced cellular health and worsened disease prognosis. Some studies have associated a lower phase angle with states of malnutrition (39); we did not see phase angle differences because the groups were matched by BMI-for-age. Furthermore, while biochemical parameters do not directly influence REE, they can serve as indirect indicators of disease. For instance, cytopenia, such as neutropenia, anemia, or thrombocytopenia, can be predictors of active infections and liver enzymes may suggest liver damage. Although the biochemical parameters are not directly related to REE, the presence of infection or diseases could increase the REE due to disease catabolism (40).

Finally, in terms of physical activity, we did not find significant differences between the two groups. However, we observed that most of the participants in the HIV-infected group were classified as not active. This means that they did not comply with the recommendation for MVPA, which could also be contributing to muscle wasting and lack of functionality (41). Regular physical activity is linked to many positive outcomes in children and adolescents. Active children have higher levels of cardiovascular fitness than their non-active counterparts. In HIV-infected children, the use of highly active antiretroviral therapy may develop unfavorable metabolic profiles that put them at risk of future cardiovascular disease, and these children may be predisposed to conditioning due to a combination of psychosociological and physiological factors, including a predisposition to a sedentary lifestyle due to the nature of their disease and/or long-term exposure to medications. In this population, exercise can help control some of the risk factors, such as cardiometabolic factors and adiposity (42). However, we know that the use of questionnaires to determine the physical activity level may have some limitations because there may be a lack of answers that require responding from memory, and exercise may be over- or under-reported.

One limitation of this study could be the equipment used to determine the REE, the Korr, which measures only VO_2_ and is self-calibrated for each participant and uses an RQ of 0.85 in a modified Weir equation to calculate REE (43). However, there are studies where this equipment has been compared to the Deltatrac Metabolic Monitor^®^ (Datex, Finland), manufactured 35 years ago, which is often considered the reference device (44–46). In this study, the validity of the calorimeter used compared with this reference calorimeter was 14 kcal/day. A small coefficient of variation (11.9%) was also observed (47).

Another limitation is that the Tanner stage was not considered in the population analyzed. Tanner stage is an additional determinant of REE, and studies have reported higher VO_2_ max in pubertal boys than in prepubertal boys. It was also higher in pubertal boys than in pubertal girls. However, the greater changes in REE and maximal aerobic power observed in boys may also be due to variations in the hormonal status and the metabolic activity of muscle tissue. Therefore, increases in physical capacities and REE during the onset of puberty indicate gender differences, which could be explained mainly by alterations in body composition in boys and girls and by changes in hormonal status in boys. Despite this, in several studies, the regression analysis of the significant determinants of REE has been controversial because the Tanner stage is not considered one of the significant variables (48, 49); nevertheless, it is plausible to consider the Tanner stage in the determination of REE. However, height remained a significant factor in the model analysis of REE, reflecting the influence of the Tanner stage. We consider this to be an important limitation of our study.

Contrarily, BIA validation studies in the pediatric population in the context of HIV have been limited in terms of comparing the technique with the dilution of isotopes and dual-energy X-ray absorptiometry. Most of the evidence is focused on predicting FFM and FM (50–52). Therefore, a limitation of BIA for predicting body composition in HIV-infected children is the absence of validation studies for the internal equations.

## 6. Conclusion

In pediatric patients with asymptomatic HIV infection, higher resting energy expenditure, lower FFM and strength, and lower dietary calorie intake were observed, despite the lack of difference in the level of physical activity.

## Data availability statement

The raw data supporting the conclusions of this article will be provided by the authors upon reasonable request.

## Ethics statement

The studies involving humans were approved by the Comité de Investigación del Instituto Nacional de Pediatría with number 2020/026. The studies were conducted in accordance with the local legislation and institutional requirements. Written informed consent for participation in this study was provided by the participants' legal guardians/next of kin.

## Author contributions

MCM-S-H, XL-L, JO-O, and IM-V: contributed to the conception and design of the study. AF-O, MCM-S-H, XL-L, BP-N, ALP-G, AG-G, LG-P, and IM-V: contributed to the acquisition of data. AF-O, MG-C, AA-N, and IM-V: contributed to the analysis and interpretation of data. AF-O and IM-V: contributed to the drafting of the article. All authors gave their final approval of the version to be submitted.
