# Supplementary material for: High resting energy expenditure, less fat-free mass, and less muscle strength in HIV-infected children: a matched, cross-sectional study
Source: Front Nutr. 2023 Sep 20;10:1220013. doi: 10.3389/fnut.2023.1220013 (PMC10548389; doi:10.3389/fnut.2023.1220013)
Supplement: Supplementary file 1 [file Data_Sheet_1.docx]

***Supplementary Material***

**High resting energy expenditure, less muscle skeletal mass and less muscle strength in HIV-infected children: A matched, cross-sectional study**

**Andrea Franco-Oliva^1,2^; Beatriz Adriana Pinzón-Navarro^3,4^; Martha Cecilia Martínez-Soto^3^; Ximena León-Lara^5^; Javier Ordoñez-Ortega^6^; Ana Pardo-Gutiérrez^1^; Martha Guevara-Cruz^4,7^; Azalia Avila-Nava^8^, Alda Daniela García-Guzmán^4,9^; Laura Guevara-Pedraza^10^; Isabel Medina-Vera^1,4, *^.**

*** Correspondence:** Isabel Medina-Vera: isabelj.medinav@gmail.com

# Supplementary Data

| Supplementary table 1. Routinary serum biochemical parameters of HIV-infected group. | |
| --- | --- |
| CD4+, *cells/mm^3^* | 779 (615.5-937) |
| CD4, *%* | 30 ± 5.7 |
| CD8+, *cells/mm^3^* | 821.6 ± 260.1 |
| CD8, *%* | 35.3 ± 4.7 |
| CD4/CD8, *ratio* | 0.85 ± 0.23 |
| Viral load, *copies/ml of blood* | <40 (n=32)  318 (74-800) (n=7) |
| Hemoglobin, *gr/dl* | 14 ± 1.3 |
| Leukocytes, *cells/mm^3^* | 6.7 ± 1.6 |
| Neutrophils, *cells/mm^3^* | 3.7 ± 1.8 |
| Lymphocytes, *cells/mm^3^* | 2.4 ± 0.4 |
| Platelet, *cells/mm^3^* | 241 (220-293) |
| Aspartate transaminase, *U/L* | 23 ± 9.9 |
| Alanine aminotransferase, *U/L* | 19.7 (14.1-29.2) |
| Alkaline phosphatase, *U/L* | 212 ± 107.3 |
| Gamma-glutamyl transferase, *U/L* | 15.8 ± 5.3 |
| Bilirubin, *mg/dL* | 0.5 (0.3-0.7) |
| Indirect bilirubin, *mg/dL* | 0.4 (0.3-0.5) |
| Albumin, *g/dL* | 4.5 ± 0.3 |
| Cholesterol*, mg/dL* | 170.8 ± 19.8 |
| LDL*, mg/dL* | 86.2 ± 14.4 |
| HDL*, mg/dL* | 45.4 ± 10.1 |
| Triglycerides*, mg/dL* | 151.5 (115.6-193.5) |
| Glucose*, mg/dL* | 92.7 ± 7.3 |
| Creatinine, *mg/dL* | 0.4 ± 0.2 |
| Glomerular filtration rate, *ml/min/1.73m^2^* | 150.4 ± 53.7 |

Data are shown as mean ± standard deviation and as median (25^th^ percentile- 75^th^ percentile).

| **Supplementary table 2. Analysis of micronutrients consumption between groups.** | | | | |
| --- | --- | --- | --- | --- |
| **Food** | **HIV-infected group n=39** | **Control group n=39** | **p** | **Recommendation** |
| Folate, mcg | 348.7 ± 146.5 | 503.3 ± 171.6 | <0.0001 | 300 (300-400) |
| Sodium, mg | 2548 (1928-3363) | 3238 (2384-4076) | 0.014 | 1800 (1800-2300) |
| Fiber, g | 12 (9-18) | 16 (13-22) | 0.079 | 31 (26-31) |
| Niacin, mg | 22.6 ± 11.2 | 27.9 ± 11.6 | 0.043 | 12 (12-14) |
| Riboflavin, mg | 1.9 ± 0.8 | 2.5± 1.3 | 0.016 | 0.9 (0.9-1) |
| Thiamine, mg | 1.2 ± 0.5 | 1.6 ± 0.5 | 0.002 | 0.9 (0.9-1) |
| Vitamin A, mcg | 456 (265-718) | 639 (489-898) | 0.025 | 600 (600-700) |
| Vitamin B6, mg | 2.0 ± 1.0 | 2.2 ± 0.9 | 0.403 | 1 (1-1.2) |
| Vitamin B12, mcg | 5.1 (3.3-6.7) | 5.8 (3.7-6.8) | 0.300 | 1.8 (1.8-2.4) |
| Vitamin C, mg | 52 (27-123) | 55 (18-132) | 0.673 | 45 (45-65) |
| Vitamin D, IU | 296.6 ± 244.2 | 296.2 ± 152 | 0.994 | 600 (600-600) |
| Vitamin E, mg | 4.6 ± 2.0 | 7.0 ± 3.6 | 0.003 | 11 (11-15) |
| Calcium, mg | 887.5 ± 454 | 1214.2 ± 484.8 | 0.004 | 1300 (1300-1300) |
| Copper, mg | 0.9 (0.7-1.2) | 1.1 (0.8-1.3) | 0.030 | 0.7 (0.7-0.9) |
| Iron, mg | 10.9 ± 3.9 | 14.4 ± 5.5 | 0.001 | 8 (8-11) |
| Magnesium, mg | 280.2 ± 111 | 324 ± 139.2 | 0.123 | 240 (240-360) |
| Phosphorus, mg | 1437.5 ± 541.3 | 1762.7 ± 690.9 | 0.012 | 1250 (1250-1250) |
| Potassium, mg | 2557.9 ± 953.4 | 2831 ± 1118.3 | 0.253 | 2500 (2300-2500) |
| Selenium, mcg | 117.7 ± 54.7 | 139.3 ± 47.5 | 0.064 | 40 (40-55) |
| Zinc, mg | 12.6 ± 7.7 | 14 ± 8.7 | 0.446 | 8 (8-9) |
| Data are shown as mean ± standard deviation and as median (25^th^ percentile- 75^th^ percentile). Student's t statistical analysis for paired samples and Wilcoxon statistical analysis. mcg: micrograms; mg: milligrams; IU: international units. | | | | |


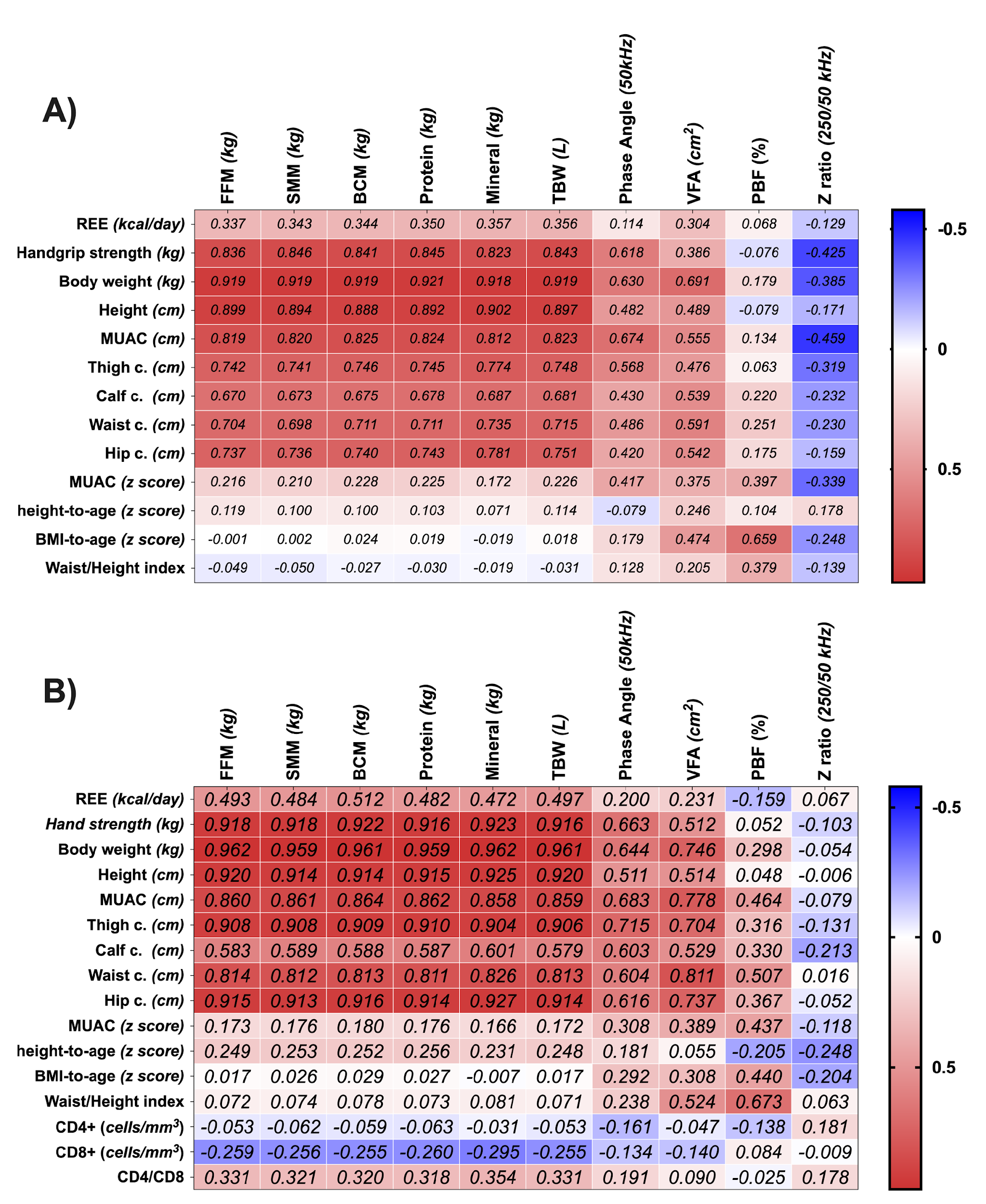


**Supplementary figure 1**. Heatmap of correlation between anthropometric indicators and BIA indicators in **A)** uninfected group and **B)** Control group.

**
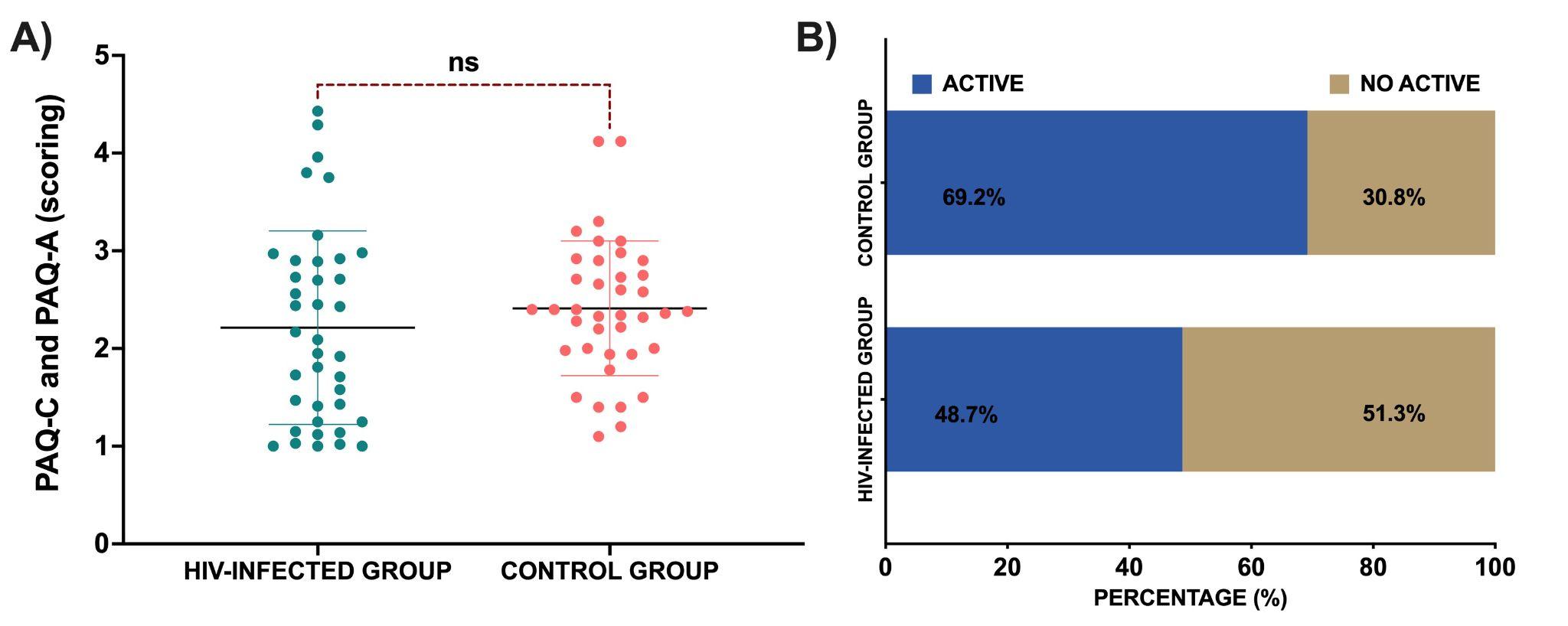
**

**Supplementary figure 2.** Physical activity. A) Scoring of physical activity, B) strata of physical activity. P.A: physical activity.
